# KCUNET: Multi-Focus Image Fusion via the Parallel Integration of KAN and Convolutional Layers

**DOI:** 10.3390/e27080785

**Published:** 2025-07-24

**Authors:** Jing Fang, Ruxian Wang, Xinglin Ning, Ruiqing Wang, Shuyun Teng, Xuran Liu, Zhipeng Zhang, Wenfeng Lu, Shaohai Hu, Jingjing Wang

**Affiliations:** 1 School of Physics and Electronics, Shandong Normal University, Jinan 250358, China; fangjing@sdnu.edu.cn (J.F.); 2023025907@stu.sdnu.edu.cn (R.W.); 2022020646@stu.sdnu.edu.cn (X.N.); 2023317010@stu.sdnu.edu.cn (R.W.); tengshuyun@sdnu.edu.cn (S.T.); 2024213130@stu.sdnu.edu.cn (X.L.); 2024317009@stu.sdnu.edu.cn (Z.Z.); 2 School of Management Engineering, Shandong Jianzhu University, Jinan 250101, China; luwenfeng@sdjzu.edu.cn; 3 Institute of Information Science, Beijing Jiaotong University, Beijing 100044, China; shhu@bjtu.edu.cn

**Keywords:** Kolmogorov–Arnold network, deep learning model, U-Net, multi-focus image fusion

## Abstract

Multi-focus image fusion (MFIF) is an image-processing method that aims to generate fully focused images by integrating source images from different focal planes. However, the defocus spread effect (DSE) often leads to blurred or jagged focus/defocus boundaries in fused images, which affects the quality of the image. To address this issue, this paper proposes a novel model that embeds the Kolmogorov–Arnold network with convolutional layers in parallel within the U-Net architecture (KCUNet). This model keeps the spatial dimensions of the feature map constant to maintain high-resolution details while progressively increasing the number of channels to capture multi-level features at the encoding stage. In addition, KCUNet incorporates a content-guided attention mechanism to enhance edge information processing, which is crucial for DSE reduction and edge preservation. The model’s performance is optimized through a hybrid loss function that evaluates in several aspects, including edge alignment, mask prediction, and image quality. Finally, comparative evaluations against 15 state-of-the-art methods demonstrate KCUNet’s superior performance in both qualitative and quantitative analyses.

## 1. Introduction

Depth of field (DOF) is defined as the distance between the nearest and furthest points that appear in focus, with objects within this range maintaining sharpness while those outside it gradually blur. In photography, the limitations of DOF prevent optical lenses from capturing images where all elements are simultaneously in focus. However, achieving a fully focused image is often desired in practice. To address this challenge, multi-focus image fusion (MFIF) has been developed as a solution. This technique combines multiple source images, each with different focus points, to produce a single image that is sharp across all focal planes, thereby enriching the scene’s information. By doing so, MFIF effectively extends the DOF of optical lenses, enabling imaging systems to overcome DOF constraints and produce higher-quality images.

MFIF algorithms have been extensively studied for decades and can generally be categorized into traditional approaches and deep learning-based approaches [1]. Traditional approaches are further divided into transform domain-based methods and spatial domain-based methods. Transform domain-based approaches involve converting the image from the spatial domain to a transform domain (e.g., wavelet transform, DCT transform, contourlet transform, etc.), extracting image features, and processing them using the characteristics of the transform domain. The final fused result is obtained by transforming the processed data back to the spatial domain through an inverse transformation. However, this method often yields unrealistic results. On the other hand, spatial domain-based approaches include three main categories: pixel-based [2,3], block-based [4,5], and region-based [6,7] methods. These methods differentiate focusing conditions at the pixel, block, or region level, respectively. Pixel-based methods are prone to mismatch issues, while block-based techniques are often sensitive to block size. Additionally, the region segmentation process in region-based methods can significantly impact their efficiency and performance.

In recent years, deep learning technology has advanced rapidly, and deep learning-based MFIF has attracted widespread attention. Numerous experimental results demonstrate that the quality of fusion outcomes achieved through deep learning significantly surpasses that of traditional techniques. Currently, MFIF methods based on deep learning include decision map-based methods [8] and end-to-end methods [9] according to the model process. The former generates a probability map, known as a decision map, by learning the features of the image, which indicates the level of pixel or region focus activity. This map is then used to determine whether to retain a region and to wait or select the original image to generate the fused image. While this approach offers flexibility in designing specific processing strategies for different focus regions, it requires additional post-processing steps, thereby increasing the computational complexity. The latter directly extracts features from the input image and merges them into an image without generating decision maps. However, the fused result has artifacts that affect fusion quality. Deep learning approaches can be broadly classified into supervised [10] and unsupervised [11] learning paradigms based on the availability of labeled training data. Supervised learning relies on annotated datasets where each input sample is paired with corresponding output labels, typically requiring substantial amounts of labeled data. However, acquiring authentic MFIF datasets for training remains challenging due to practical imaging constraints. While unsupervised learning eliminates the need for manual labeling by extracting features directly from unlabeled data, this approach often suffers from high computational demands and inconsistent fusion performance, potentially leading to detail loss or artifact generation. Given these considerations, we propose a supervised deep learning model capable of generating both decision maps and fusion results for use in MFIF.

In 2015, O. Ronneberger et al. [12] introduced the U-Net model, which has since become widely adopted in image processing and spawned numerous variants. Cicek et al. [13] adapted the U-Net architecture for 3D image segmentation, significantly improving 3D-imaging accuracy. Zhou et al. [14] further advanced the architecture by developing U-Net++, which integrates nested structures and dense feature fusion mechanisms. Recent advances in transformer technology have led to novel hybrid architectures that combine U-Net’s precise localization capabilities with transformers’ ability to capture long-range dependencies. Cao et al. [15] proposed Swin-UNet, a medical image segmentation network that incorporates the Swin transformer architecture. Currently, the Kolmogorov–Arnold network (KAN) model is being used to enhance the ability of models to capture complex features and patterns due to its unique structural design. By leveraging its superior nonlinear modeling capabilities, KAN can effectively capture intricate relationships within images, enabling more efficient global feature extraction.

Building upon these advancements, this paper proposes KCUNet, an innovative U-Net variant that embeds the KAN model in parallel with a convolutional layer into the encoder of the UNet network. The KAN model, which has attracted significant research attention this year, combines an attention mechanism that can better capture key information and a kernel approach that enhances the nonlinear representation of the model. In the KCUNet architecture, while convolutional layers extract fundamental features, parallel KAN layers provide complementary contextual understanding, collectively strengthening the model’s ability to handle complex visual scenes. In addition, the traditional U-Net network extracts features by downsampling and upsampling, but continuous downsampling loses some important spatial information and details, leading to blurring of image boundaries and details. Therefore, the coding part of this paper no longer reduces the feature map size. The principal contributions of this work are as follows.

- This paper proposes a novel MFIF network based U-Net called KCUNet, which a parallel KAN layer alongside convolutional layers in the encoder, maintaining consistent spatial dimensions of feature maps while progressively increasing channel depth to capture multi-scale features effectively.
- This paper designs a spatial–channel guided attention network (S–CGANet), which boosts edge preservation by combining Laplace pyramid-based high-frequency feature extraction with channel guided attention (CGA), refining boundary localization and reducing defocus effects in image fusion.
- This paper designs a hybrid loss function that comprehensively considers the performance of the model in several aspects including edge alignment, mask prediction and fused images.
- This paper demonstrates the performance of KCUNet through extensive comparative experiments and ablation studies. Compared with 15 state-of-the-art (SOTA) methods, KCUNet shows superior performance in focal region detection, visual perception analysis and quantitative scoring.

The remainder of this paper is structured as follows. A review of some related work is provided in Section 2. The proposed model structure and loss function are described in detail in Section 3. Experimental results are presented and discussed analytically in Section 4. The paper is concluded in Section 5.

## 2. Related Work

### 2.1. Traditional MFIF Methods

In the 1980s, MFIF technology began to emerge, with traditional methods broadly categorized into transform domain-based and spatial domain-based approaches. During the early development of transform domain methodology, Burt et al. [16] pioneered the application of the Laplace pyramid approach to MFIF. This method involved recursively applying Gaussian filtering and downsampling to decompose an image into multiple layers of varying resolutions, enabling multi-scale image analysis. Subsequently, Toet et al. [17] introduced the ratio of low-pass pyramid (RP)-based method, which utilized the ratio between low-pass pyramids of two images to determine fusion weights. The advent of the wavelet transform later led to the development of various wavelet-based methods. Lewis et al. [18] proposed a technique based on the dual-tree complex wavelet transform (DTCWT), capable of capturing both magnitude and phase information to better preserve local features and edge details. Zhang et al. [19] introduced the non-subsampled wavelet transform (NSCT) method, which avoided information loss during image decomposition and reconstruction by eliminating subsampling. Yang et al. [20] incorporated sparse representations into image integration for the first time. He et al. [21] later proposed guided image filtering (GFF), a novel filtering technique that leverages a guiding image to preserve edge and texture information while effectively suppressing noise.

To address the limitations of transform-domain methods in image fusion, researchers have developed spatial-domain approaches. Li et al. [6] introduced a spatial frequency (SF)-based method, which evaluates image sharpness by analyzing spatial frequency information, allowing the identification of focus and defocus regions in images. Further advancing the field, Li et al. [4] proposed an MFIF method incorporating region segmentation, which effectively balances local details and global features by partitioning the image into distinct regions. Zhang et al. [22] developed a boundary detection-based MFIF technique, employing a novel multi-scale morphological focus metric to extract boundary information and guide the fusion process. Panigrahy et al. [23] introduced a parameter-adaptive method that utilizes fractal dimension as a feature metric, dynamically adjusting the parameters of a two-channel impulse-coupled neural network to optimize fusion performance. Additionally, Kurban [24] proposed the Gaussian of differences (GD), a computationally efficient pixel-based framework for general image fusion. This approach enhances edge preservation through first-order derivative edge detection while incorporating neighborhood pixel contributions via Gaussian filtering. To ensure adaptability across diverse images, the method employs a pattern search algorithm for automatic parameter optimization.

### 2.2. Deep Learning-Based Methods

Recent years have witnessed the emergence of numerous deep learning-based algorithms in image fusion. In 2017, Liu et al. [8] pioneered the application of deep learning in MFIF by proposing a CNN-based model that integrates region-blocking techniques to guide activity level measurement and fusion rule generation. Zhang et al. [25] later introduced a universal end-to-end model (IFCNN), capable of handling various fusion tasks beyond MFIF. This approach directly learns fused images from input data without requiring complex post-processing. Ma et al. [11] proposed an unsupervised deep learning model (SESF-Fuse), which extracts deep features directly from the input image by training the encoder-decoder network in an unsupervised manner, which is then used for image fusion. Similarly, Jung et al. [26] proposed a model that handles all fusion stages comprehensively. This method leverages structural tensor representations of multi-channel image contrast as the basis for an unsupervised loss function, eliminating dependence on labeled data while overcoming the computational limitations of traditional approaches. Yan et al. [27] introduced a feed-forward fully convolutional network (MFNet) capable of processing variable-sized images. This model employs structural similarity (SSIM) as its loss function to optimize output quality. Ma et al. [28] investigated an end-to-end (MFIF) approach employing multi-scale generative adversarial networks (MsGAN). In this framework, source images are decomposed into multi-scale sub-images, which undergo processing through depth-variant GANs for feature extraction. The fused image is subsequently reconstructed via inverse wavelet transform, utilizing CNN-based multi-scale decomposition to optimize detail preservation. More recently, Cheng et al. [29] presented a framework (MUFusion) that adaptively refines fusion performance by incorporating intermediate training results as additional guidance, thereby enhancing the final output through multi-stage feature learning.

### 2.3. Defocus Spread Effect (DSE)

**Figure 1:**
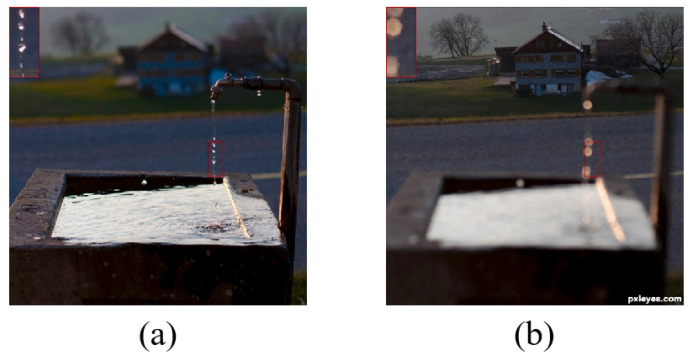
(**a**) No DSE and (**b**) with DSE. The red box in the upper left corner shows a zoomed-in view of the water droplet.

DSE is a prevalent issue that can affect the quality of image fusion. It is a fact that focused and defocused regions are not always separated by a clear boundary. When the image’s foreground is sharply focused and the background is blurred, the focused foreground is not affected by the background out of focus, and then the focus/defocus boundary (FDB) is a sharp outline, as shown in Figure 1a. But if the focus is reversed, the defocused foreground will penetrate into the background, then the FDB is an indeterminate region, as shown in Figure 1b. Therefore, how to avoid DSE is one of the main research directions at present.

In previous studies, researchers have explored various approaches to address the DSE in image fusion. Ma et al. [30] proposed a model to simulate DSE and generated training data to capture the differences in DSE when either the foreground or background is defocused. They also introduced a cascade-based boundary-aware convolutional network (MMF-Net), which refines fusion results along the FDB by generating fusion bootstrap maps through two sub-networks. Xu et al. [31] employed a gradient ascent technique to enhance the structural similarity index of fused images, ensuring that each segment closely matches its corresponding region in the source images, thereby reducing the effect of DSE. Wang et al. [32] developed MFIF-GAN, a network that generates focus maps with slightly expanded foreground regions to counteract the effects of DSE. Unlike the above methods, we use the Laplace pyramid to identify and extract high-frequency features, including edges and contours of the image. Additionally, the CGA module is employed to augment the model’s focus on edge information and retain the edge information at different levels, effectively mitigating DSE. Further details are discussed in Section 3.2.2.

### 2.4. Kolmogorov–Arnold Network (KAN)

Recent advancements in neural network architectures have led to the development of innovative models that prioritize both interpretability and performance. Among these, KAN has emerged as a promising approach, attracting significant attention due to its enhanced interpretability and efficient data-fitting capabilities with fewer parameters.

Drawing on the KAN representation theorem, MIT researchers developed KAN as an innovative network architecture [33]. The architecture’s fundamental innovation lies in its departure from conventional neural network design through the replacement of traditional weight parameters and the repositioning of activation functions. In contrast to standard multi-layer perceptrons (MLPs), KAN places learnable activation functions on the ‘edges’ of the network instead of using fixed activation functions at the nodes. Specifically, KAN eliminates fixed activation functions entirely, instead implementing them through learnable univariate functions. This architectural choice endows KAN with superior computational efficiency, accuracy, and interpretability compared to MLPs. The training process of KAN involves parameter updates through loss function computation and gradient backpropagation. This training framework enables KAN to autonomously determine optimal univariate functions that most effectively approximate the target function. Furthermore, KAN demonstrates greater parameter efficiency by eliminating the extensive weight parameters characteristic of traditional MLPs, achieving complex nonlinear transformations through fewer spline function parameters instead.

Building upon this foundation, we integrate KAN with widely used CNN and U-Net architectures, seeking to further investigate KAN’s efficacy and potential advantages in MFIF tasks.

## 3. Proposed Method

This section first provides a comprehensive overview of KCUNet, the proposed framework in this study. Subsequently, it elaborates on the network’s key components and the employed loss function in greater detail.

### 3.1. Problem Formulation

As mentioned in the previous section, MFIF aims at fusing multiple source images $\left\{I_{1},I_{2},\ldots ,I_{N}\right\}$ into one fully focused image *F*. For a typical dual image fusion scene, the process can be mathematically expressed as follows:

(1) $$F=I_{A}\times DM+I_{B}\times \left(1-DM\right),$$

where $I_{A}$ and $I_{B}\in R^{C\times H\times W}$ denote the foreground and background focus images, respectively (*C*, *H*, and *W* are the number of channels, height, and width of the image, respectively). $DM\in {\left[0,1\right]}^{1\times H\times W}$ is a decision map that identifies the focus region in $I_{A}$. The ideal DM should satisfy the following:

(2) $$\mathrm{DM}\left(x,y\right)=\left\{\begin{array}{c} 1,I_{A}\left(x,y\right)\;\mathrm{is}\;\mathrm{in}\;\mathrm{focus}\;\\ 0,I_{B}\left(x,y\right)\;\mathrm{is}\;\mathrm{in}\;\mathrm{focus}\; \end{array}.\right.$$

Here, the accuracy of DM directly affects the quality of image fusion. Generating an accurate DM requires multi-scale feature analysis, while preserving clear boundaries depends on a precise DM as a foundation. To address these challenges, we propose KCUNet, a parallel KAN-convolutional architecture that synergistically resolves these key issues. By integrating local detail features with global contextual information, the network ensures accurate capture and preservation of critical details across different scales, thereby enhancing both the visual quality and informational integrity of the fused results.

### 3.2. Overview

**Figure 2:**
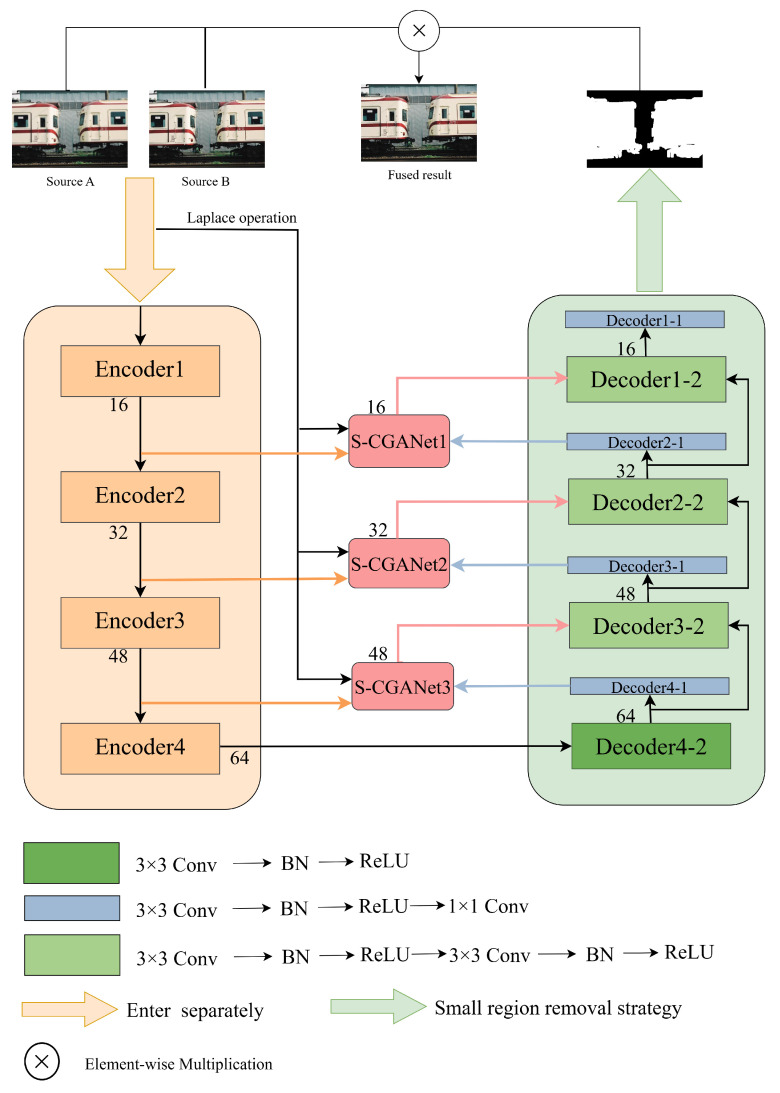
Overall integration strategy of KCUNet. The network takes as input a pair of source images in the RGB format, where Source A captures the foreground in sharp focus, while Source B highlights the background with clarity. Conv, BN, and ReLU represent the convolutional layer, batchnorm layer, and rectified linear unit, respectively. The number following each layer indicates its output channels.

As shown in Figure 2, this paper proposes KCUNet, a novel MFIF network that embeds KAN with convolutional layers in parallel within a U-Net framework. The architecture consists of three primary components: an encoding stage, a decoding stage, and an attention module.

The encoding stage comprises four encoders, each containing parallel–connected KAN and convolutional layers. Differing from the traditional U-Net architecture, KCUNet maintains the spatial sizes of feature maps throughout the encoding stage while incrementally increasing the channel count at high resolution. This approach enables each encoding layer to learn richer feature representations at consistent spatial dimensions. With increasing network depth, the expanding channel capacity allows the model to capture increasingly detailed features across different levels, thereby supplying more comprehensive feature information for subsequent fusion processes. In the decoding stage, an inverse strategy is adopted. Specifically, the channel count is progressively reduced while preserving the feature map dimensions. This design enables the model to more attentively process and integrate key features from the encoder while simultaneously reducing computational complexity and parameter volume.

In addition, the decoding part combines the edge information extracted by the attention module for feature enhancement and the Decoder x-1 layer to convert the feature map into a binary image. Specifically, the output feature maps of the encoder are fused with the enhanced feature maps from the attention module and the source image processed by the Laplace operation. That is, the feature map from Encoder 4 undergoes channel reduction via Decoder 4-2, and then is converted into a binary image by Decoder 4-1 and fed into the attention module. The attention module combined this output with the features extracted by Encoder 3 and the source image processed by Laplace operation, and then passed them to Decoder 3-2. The process is repeated three times until Decoder 1-1 produces the preliminary decision map. Finally, a small-area removal strategy is applied to generate the ultimate decision map, which is then used to generate the fusion result according to Equation (1). Table 1 shows the specifications of each module.

#### 3.2.1. Encoding Component

**Figure 3:**
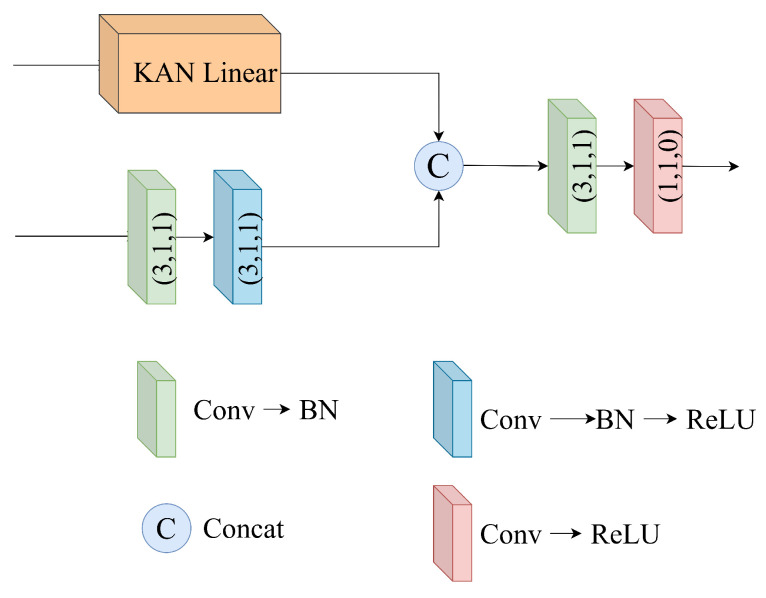
Detailed architecture of the encoder block. The kernel, stride, and padding sizes of the convolutional layer are provided by the array in brackets in each block.

The encoder consists of parallel–connected KAN layers and convolutional layers. While convolutional layers exhibit robustness to variations in object location and scale within the image due to their translation-invariant nature, they struggle with global information processing because of their reliance on local receptive fields when processing the image. In contrast, KAN leverages its nonlinear modeling capability to capture complex nonlinear relationships in images, enabling effective extraction of global features. Specifically, KAN extracts each channel’s information pixel by pixel and converts it to 1D data, which is then processed using the KAN layer and restored to 2D. By combining convolutional layers and KAN layers in a parallel dual-channel structure, the model retains both local feature sensitivity and global information awareness. As illustrated in Figure 3, the convolutional layer consists of two cascaded 3 × 3 convolutions. The features extracted from this layer are concatenated with those from the KAN layer along the channel dimension. The merged features are then further processed using a 3 × 3 convolution followed by a 1 × 1 convolution for refined feature extraction.

#### 3.2.2. Attention Module

This section further elaborates on the principle of S–CGANet. As a core component of the KCUNet architecture, the S–CGANet module exhibits robust edge-guidance capabilities and operates across multiple levels. It extracts features from low to high levels while enhancing the model’s attention to edge-related information. The module primarily employs the Laplace pyramid method to capture high-frequency variations in images and utilizes the CGA module to precisely focus on dispersion boundaries. This approach significantly improves the accuracy and robustness of feature extraction.

The CGA module, introduced in DEA-Net [34], is an innovative attention mechanism that generates a unique spatial importance map (SIM) for each channel in a CNN. Its key strength lies in leveraging input feature information to guide SIM generation, enabling the model to more accurately detect and emphasize feature-relevant regions in every channel. This mechanism first constructs an initial attention map by applying operations such as global maximum pooling and global average pooling across channel and spatial dimensions. The channel and spatial attention components are then combined through addition to produce a preliminary SIM. Finally, the CGA refines this coarse SIM for each channel using channel shuffling and grouped convolution, yielding channel-specific SIMs that enhance the model’s ability to focus on the most discriminative feature information.

The Laplace pyramid method builds an efficient multi-scale representation by progressively reducing image resolution and storing inter-layer differences, thereby improving both efficiency and accuracy in image analysis. When processing source image pairs, the Laplace pyramid preserves critical edge information. Among them, the Laplace operator, as a second-order derivative edge detection tool, captures high-frequency features, such as edges and contours, by analyzing second-order variations in the input image. These extracted high-frequency features enable the identification of FDB and effectively mitigating DSE. It should be noted that while the Laplace operator exhibits sensitivity to high-frequency noise, our design effectively addresses this limitation through two key mechanisms. Firstly, the multi-scale decomposition framework of the Laplace pyramid inherently suppresses high-frequency noise at coarse scales while preserving meaningful edge information. Secondly, the CGA module dynamically attenuates noise-dominated feature channels through its channel-adaptive SIMs.

**Figure 4:**
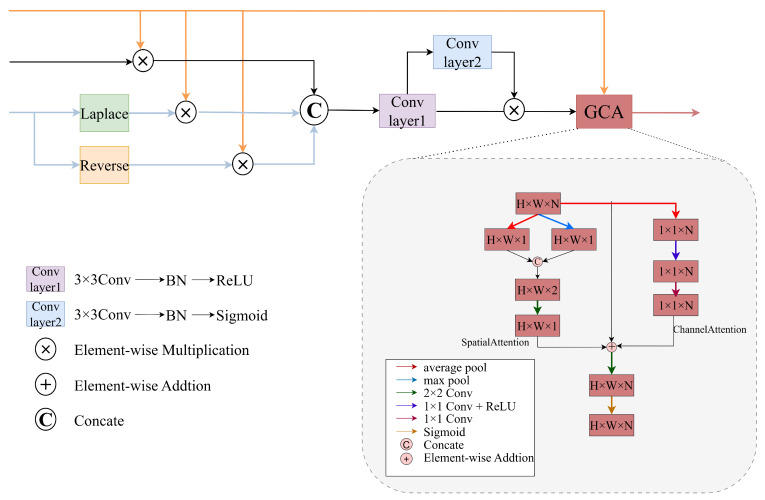
The architecture of the S–CGANet block. Laplace denotes the application of the Laplace operator to generate a bounded attention map, and Reverse denotes the generation of reverse attention by a one-subtraction operation. H, W, and N denote the height, width, and channel counts of the input. Orange arrows denote feature maps from the encoder, black arrows represent high-frequency features extracted by the Laplace pyramid, and blue arrows indicate predicted features from the decoder.

As illustrated in Figure 4, the S–CGANet module enhances the model’s ability to focus on edge-related information by integrating diverse features and leveraging the robust edge-guidance capability of the CGA. This mechanism preserves critical high-frequency details across different layers, particularly the edge information that tends to diminish as network depth increases. Specifically, the S–CGANet module at each layer processes three inputs: the embedded features from the encoder, the predicted features from the higher layers of the decoder, and the high-frequency features extracted by the Laplace operator. The predicted features at the decoder’s output generate an inverse attention map through a single subtraction operation, while the boundary attention map is produced by applying the Laplace operator. These two attention maps, along with the high-frequency features extracted by the Laplace operator, are fed into the CGA attention module after undergoing element-wise multiplication with the embedded features from the current encoder. This integration occurs through sequential operations of concatenation, convolution, and element-wise multiplication. Consequently, the S–CGANet module achieves more precise localization of defocus boundaries, significantly improving the accuracy and robustness of feature extraction. This approach retains more edge features while mitigating the impact of DSE on the MFIF task.

### 3.3. Loss Function

In deep learning, the loss function plays a crucial role in determining model performance, as it guides the learning direction of the model and drives the model’s predictions toward ground truth values. To comprehensively consider the effectiveness of KCUNet, we propose a hybrid loss function $L_{total}$, which guides the training of the model by combining different loss components, while constraining the output decision map and fusion results. Specifically, it consists of three main components, each of which is optimized for a different aspect of model performance, as defined below:

(3) $$L_{total}=\lambda_{1}L_{EA}+\lambda_{2}L_{Mask}+\lambda_{3}L_{Q_{g}},$$

where $\lambda_{i}$ is the hyperparameter for adjusting the influence of each loss component on the overall loss. This design allows flexibility in adjusting how much attention the model pays to different performance metrics during training, which will be set to 1 in this paper.

#### 3.3.1. Edge Alignment Loss


(4) $$L_{EA}=\alpha_{1}L_{BCE}+\alpha_{2}L_{Dice}.$$

This part of the loss function focuses on the performance of KCUNet in terms of edge alignment. In deep networks, some important detailed information may be lost as the depth of the network increases. We apply the loss function on multiple decoding layers and use this loss function to measure the difference between the predicted probability distributions and the true labels at different levels, which makes the model focus on the detailed information at each stage and reduces the information loss. It consists of $L_{BCE}$ and $L_{Dice}$, two sub-losses corresponding to the bounded cross-entropy loss and the Dice loss. The weights of $\alpha_{i}$ are controlled and are set to 0.5 in this paper. The combination of these two losses allows the model to be more accurate in predicting edges while maintaining the consistency of the region. The weights of this part of the loss are controlled by $\lambda_{1}$.

$L_{Dice}$ is defined in Equation (3) as the loss function in semantic segmentation:

(5) $$L_{Dice}=1-\frac{2\sum_{i}^{N_{P}}p_{i}g_{i}+1}{\sum_{i}^{N_{P}}p_{i}^{2}+\sum_{i}^{N_{P}}g_{i}^{2}+1},$$

which sums the predicted binary segmentation map $p_{i}$ and the ground truth map $g_{i}$ over $N_{p}$ pixels. The addition of 1 helps to address the issue of gradient loss. This loss function measures the agreement between the predicted decision map and the true values, i.e., the segmentation accuracy of the target region. By comparing their similarity, it constrains the model parameters and improves the network’s ability to identify different targets.

$L_{BCE}$ is a classical binary loss defined as follows:

(6) $$L_{\mathrm{BCE}}=\frac{-\sum_{i,j}^{H,W}\left[G_{\left(i,j\right)}\ln\left({\mathrm{DM}}_{\left(i,j\right)}\right)+\left(1-G_{\left(i,j\right)}\right)\ln\left(1-{\mathrm{DM}}_{\left(i,j\right)}\right)\right]}{n},$$

where $G_{\left(i,j\right)}$ represents the ground truth binary mask, and $DM_{\left(i,j\right)}$ is the predicted decision map. $L_{BCE}$ can access the correct classification of each pixel and improves the network’s ability to distinguish between sharp and blurred pixels.

#### 3.3.2. Mask Loss


(7) $$L_{Mask}=L_{Dice}+L_{SSIM}.$$

The mask loss evaluates the model’s performance in mask prediction by measuring the discrepancy between the decision map and the ground truth labels. This loss component combines Dice loss and structural similarity index (SSIM) loss to enhance both accuracy and structural preservation in mask segmentation. The parameter $\lambda_{2}$ regulates the weight of the mask loss.

The $L_{SSIM}$ loss function constrains the brightness, contrast, and structural metrics of the image, ensuring that the fusion result maintains greater similarity with the input image. It is computed using the following formula:

(8) $$L_{SSIM}\left(I_{A},I_{B},F\right)=1-\frac{1}{N_{P}}\sum_{\omega =1}^{N_{P}}S\left(I_{A},I_{B},F|\omega \right),$$

where $I_{A}$ and $I_{B}$ serve as the input images, with F being the fused result, $N_{P}$ represents the total count of sliding windows, ω denotes a specific local window, and *S* is defined as follows:

(9) $$\begin{array}{c} S\left(I_{A},I_{B},F|\omega \right)=\left\{\begin{array}{c} SSIM\left(I_{A},F|\omega \right)ifstd\left(I_{A},|\omega \right)\geq std\left(I_{B},|\omega \right)\\ SSIM\left(I_{B},F|\omega \right)otherwise \end{array}\right. \end{array},$$

where $SSIM(I_{A},F|\omega )$ refers to the SSIM index calculated between image patch $I_{A}$ and *F* within a local window ω, ω=11×11 in this paper, and $std(I_{A},|\omega )$ represents the standard deviation of image patch $I_{A}$ within the local window ω.

#### 3.3.3. Quality Loss

$L_{Qg}$ is derived from the gradient-aware loss function in GACN [9], enhancing gradient preservation in the fusion output by regulating edge information retention at the pixel level. For detailed formulation and parameters, please consult the original GACN reference. The weights are controlled by $\lambda_{3}$.

## 4. Experiment

### 4.1. Execution Details

**Figure 5:**
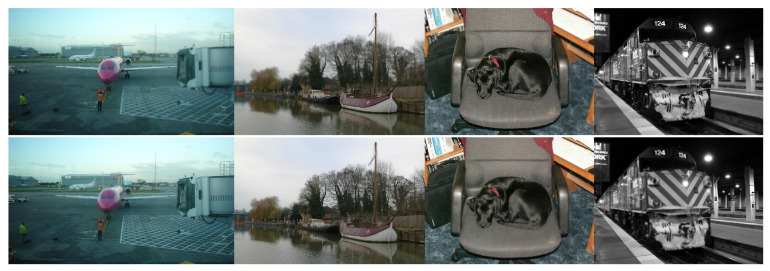
Training examples derived from the VOC2012 dataset. The top row displays the image with near-focus, while the bottom row shows the image with far-focus.

Dataset selection plays a crucial role in model performance, yet acquiring real MFIF datasets for training remains challenging due to practical shooting constraints. To address this, we employ the VOC 2012 dataset [35], which comprises 2913 images with corresponding segmentation masks. We synthesize MFIF training data by applying Gaussian filtering to create two variants of each image: one with blurred foreground and another with blurred background. The α-matte boundary defocus spread model [30] is then used to simulate the DSE. The synthesized dataset is split in a 7:3 ratio, yielding 2016 image pairs for training and 897 pairs for validation. Figure 5 illustrates samples from our synthetic MFIF dataset.

During the training process, the input image size is first resized to a uniform 256 × 256 pixels to enhance the model’s computational efficiency. Next, the image is randomly cropped to 156 × 156 pixels to simulate diverse scenarios and increase data diversity. Additionally, the input data undergo random horizontal and vertical flipping, as well as random rotation at angles selected from 0, 30, 60, 90, 120, 150, 180, 210, 240, 270, 300, and 330 degrees. This helps the model adapt to varying angles and orientations, thereby improving its generalization capability. To further enhance robustness, data augmentation techniques such as random blurring, random erasure, random offset, and Gaussian noise addition are applied. These operations strengthen the model’s ability to handle various image disturbances, ensuring stable performance even with incomplete information or in complex environments. In contrast, no preprocessing is performed during the validation phase to accurately simulate the model’s performance on real-world datasets.

KCUNet was trained using the PyTorch framework with the following configurations: an initial learning rate of 0.0001, 50 epochs, and a learning rate reduction by a factor of 0.8 every two epochs. The weight decay was set to 0.0001, and the Adam optimizer was employed to adaptively adjust the learning rate for each parameter. A batch size of eight was used during training. The model was trained and tested on an NVIDIA Quadro P5000 GPU with 16GB of memory.

### 4.2. Test Datasets

For the MFIF task, the test datasets consist of both real-world and synthetic images. This study evaluates model performance using the Lytro dataset and the MFFW dataset for real image fusion, while the MFI-WHU dataset is employed to assess synthetic image fusion performance.

The Lytro camera is a light-field imaging device that records not only light intensity but also the direction of light. This unique capability enables post-capture focus adjustment in the resulting images. The Lytro dataset [36] contains 20 image pairs, each with dimensions of 520 × 520 pixels, all captured by the Lytro light-field camera under identical scene conditions but with varying focal points.

The MFFW dataset, specifically designed for MFIF, contains 12 real multi-focus image pairs exhibiting significant DSE. These images are from real-world scenes with complex backgrounds and variable lighting conditions, presenting a rigorous challenge for fusion algorithms.

For synthetic image evaluation, the MFI-WHU dataset [37] is utilized. Constructed by Zhang et al., this dataset derives from the COCO datasets [38,39], where 120 clear images were selected and processed using Gaussian blur and manual decision maps to generate multi-focus pairs. From these, 18 image pairs are chosen for testing in this study.

### 4.3. Assessment Methods

Assessing the quality of image fusion in the MFIF can be categorized into two approaches: objective and subjective methods. The objective approach, known as quantitative evaluation, employs evaluation metrics to assess fusion quality. These metrics fall into four main categories: information theory-based, image feature-based, structural similarity-based, and human perception-inspired metrics [40]. Each category provides a distinct perspective for evaluating fusion algorithm performance. The subjective approach, also referred to as qualitative evaluation, relies on human observers to visually inspect fused images and make judgments based on personal perception. While straightforward, this method is inherently subjective and prone to bias. Consequently, subjective evaluation is typically used in conjunction with objective methods.

We select eight fusion metrics from four categories to assess the performance of KCUNet. Theory-based information, including MI [41] and NCIE [42], human perception-inspired fusion metrics, including $Q_{CB}$ [43], image structural similarity-based metrics, including SSIM [44], image feature-based metrics, including $Q_{M}$ [45], $Q_{G}$ [46], GLD [47], and MSD [47]. For the metrics, higher values indicate higher performance. The objective evaluation provides a direct numerical assessment of the model’s fusion efficiency. To ensure fairness, default parameters are applied consistently across all metrics. All calculations are performed using MATLAB R2023a.

### 4.4. Comparison with SOTA Methods

We conduct a comprehensive comparison between KCUNet and 15 SOTA methods, encompassing both traditional and deep learning-based approaches. Traditional methods include BFMF [22] and DCT-EOL [48], deep learning based methods include CNN [8], ECNN [49], IFCNN [25], DRPL [10], MFF-Net [30], U2Fusion [50], MFF-GAN [37], MFIF-GAN [32], SESF [11], SwinFusion [51], MiT [52], ZMFF [53], and FusionDiff [54].

#### 4.4.1. Subjective Analyses

**Figure 6:**
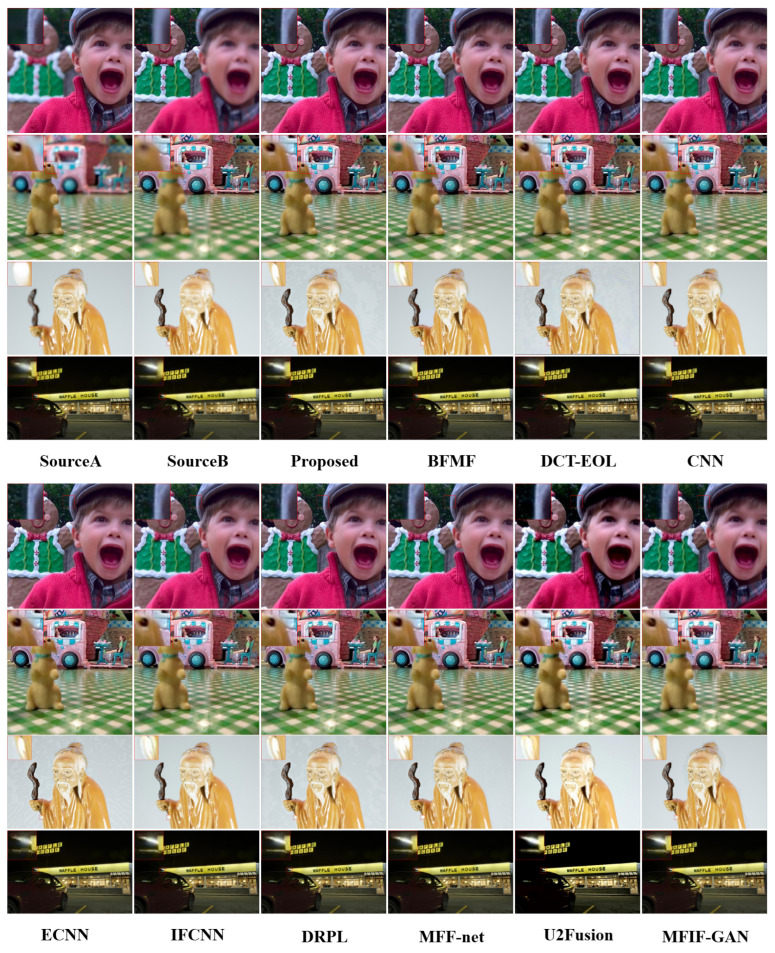

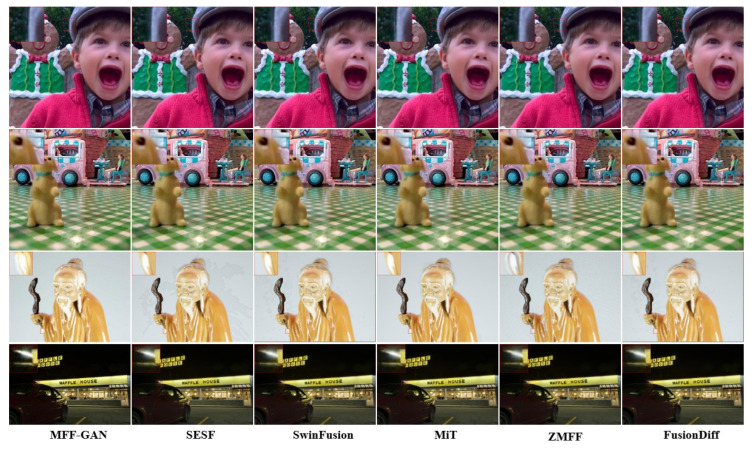
The fusion results for all algorithms with detail enlargement on Lytro (3rd), Lytro (17th), MFFW (4th), and MFI-WHU (8th).

For visual comparison, representative images from three benchmark datasets were selected: the 3rd and 17th images from the Lytro dataset, the 4th image from the MFFW dataset, and the 8th image from the MFI-WHU dataset. Figure 6 presents the comparative visual results between KCUNet and the 15 state-of-the-art methods, demonstrating the performance differences across different fusion scenarios.

To better visualize the subtle differences between the various methods, we select specific local regions from the images and enlarge them using bilinear interpolation. A smaller red rectangle in each figure marks the selected region for magnification, while the enlarged view is displayed in the upper left corner of the image, corresponding to the larger red rectangle. Source A and Source B represent the near-focus and far-focus images, respectively, while the remaining images display fusion results from different models, with their corresponding method names labeled below. In the third image of the Lytro dataset, the edges in the BFMF result appear blurred, while the hat edges in the DCT-EOL, DRPL, MFIF-GAN, and FusionDiff results exhibit varying degrees of artifacts. In the seventeenth image, the BFMF method causes the doll’s eyes to appear blurred and discolored, whereas the DCT-EOL, FusionDiff, ECNN, and Swin Fusion methods introduce jagged edges, and the DRPL method produces artifacts. For the fourth image in the MFFW dataset, significant diffusion blurring is observed at the old man’s sleeve in the results of BFMF, CBF, DRPL, and NSCT-SR, while IFCNN, MFF-GAN, U2Fusion, and ZMFF exhibit severe diffusion blurring. Additionally, MiT, U2Fusion, and MMFNet display noticeable ghosting artifacts. In the eighth image of the MFI-WHU dataset, BFMF, CNN, ECNN, and SESF show severe blurring around the light. Furthermore, the U2Fusion method produces fusion results with significant color discrepancies.

The above comparison demonstrates that KCUNet exhibits significant advantages in visual performance, particularly in preserving edge details and color accuracy, resulting in more natural and realistic fused images. Consequently, KCUNet excels in addressing DSE and offers an effective solution for MFIF.

#### 4.4.2. Objective Analyses

Objectively, Table 2 provides the average measurement results for all images in the testing dataset. Compared to other SOTA methods, KCUNet obtains the highest scores on seven out of eight evaluation metrics for both the Lytro and MFFW datasets, while closely approaching the highest score on the remaining metric. On the MFI-WHU dataset, KCUNet ranks first in five out of eight metrics. Both Lytro and MFFW are real images captured by a camera, and in particular, there is significant DSF in the MFFW dataset, and KCUNet’s good performance in both datasets suggests the effective mitigation of DSE. Among the specific evaluation metrics, it is noteworthy that KCUNet achieves the highest scores on MI and NCIE, which indicates that the fusion results retain a substantial amount of information from the source image. The highest score for $Q_{G}$ indicates that the fusion results successfully inherit the rich detail and edge information from the source image. The highest scores for $Q_{M}$ and MSD indicate KCUNet effectively maintains multiscale structural information, further validating its ability to retain hierarchical features from source images. Overall, the highest and near-highest values for the other evaluation metrics further emphasize that the method produces fusion results with high similarity to the source image with fewer artifacts. However, there remains room for improvement in $Q_{CB}$ and GLD, likely due to a trade-off where enhancing fine details slightly compromises image smoothness, leading to minor visual distortions. This limitation will be thoroughly investigated in future work to further enhance fusion quality.

In addition, Table 2 presents the average fusion time for each method. While KCUNet demonstrates superior performance in fusion quality, its computational efficiency does not show a significant advantage over most competing methods. This limitation likely stems from the KAN model’s dense connection architecture, which increases computational complexity and reduces processing speed.

### 4.5. Ablation Experiments

#### 4.5.1. Effect of KAN

**Figure 7:**
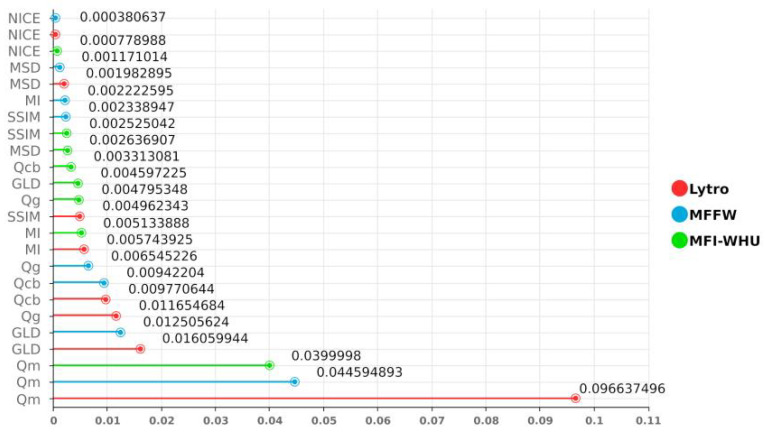
The differences in the experimental results. Red represents the Lytro dataset, blue represents the MFFW dataset, and green represents the MFI-WHU dataset.

To validate the critical role of the KAN layer, we conduct comparative experiments between KCUNet and a purely convolutional layer encoder across three benchmark datasets (Lytro, MFFW, and MFI-WHU). As evidenced by Table 3, KCUNet’s fusion results consistently outperform the KAN-free variant across all eight evaluation metrics. Among them, the differences in $Q_{M}$, GLD, $Q_{G}$, and $Q_{CB}$ are more obvious, which indicates that the KAN layer has a better ability to extract edge and detail information and can extract more multi-scale information. Figure 7 demonstrates the differences in the experimental results. It can be seen that the KAN structure can better capture the complex transition features of DSE due to its nonlinear modeling capability. Additionally, by processing channel information at the pixel level, KAN compensates for traditional convolution’s limitations in global feature extraction. The parallel design of KAN and convolutional layers further achieves a balance between local feature sensitivity and global context awareness. While KAN increases computation time by approximately 3–4 times, the performance gains outweigh this cost. Future research could extend the KAN structure to other image processing tasks requiring fine boundary processing, particularly in scenarios where preserving both local details and global structure is crucial.

#### 4.5.2. Impact of Encoder and Attention Modules

KCUNet primarily consists of three components: the encoding part, the decoding part, and the attention part. To investigate the impact of the designed encoder and attention module, we compare image fusion results using KCUNet with the following three approaches:

(i) Image fusion using only the decoder. This evaluates the fused image quality without the assistance of the encoder and attention module.

(ii) Image fusion using both the encoder and decoder. By integrating the encoder with the decoder, the role of the proposed encoder can be assessed.

(iii) Image fusion using both the attention module and decoder. This combines the attention module with the decoder to verify the contribution of the attention module.

**Figure 8:**
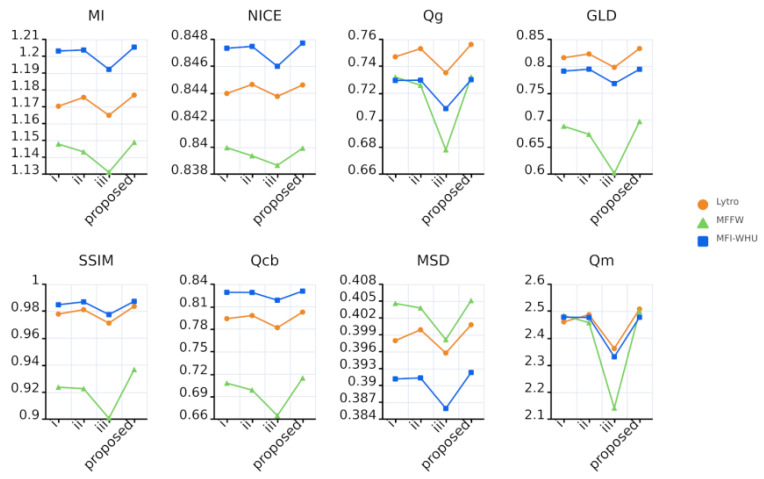
The values of the eight fusion metrics when fusing the different datasets using KCUNet and each of these three approaches.

Figure 8 shows the values of the eight fusion metrics obtained when fusing the Lytro, MFFW and MFI-WHU datasets using KCUNet and these approaches. Where circles represent the Lytro dataset, triangles represent the MFFW dataset, and rectangles represent the MFI-WHU dataset. As shown in the figure, most metrics exhibit lower values when only the decoder is employed, suggesting that the decoder alone processes limited information in the fusion results. After incorporating the encoder, the metric values increase, demonstrating that the encoder enhances feature extraction and thereby improves the information content of the fused images. However, when the attention module is used alongside the decoder, all metric values reach their minimum, indicating that the attention module fails to correctly learn which features are important. In contrast, KCUNet achieves the highest values for most fusion metrics, confirming that the model’s design is well-justified.

#### 4.5.3. Effects of Different Loss Functions

**Figure 9:**
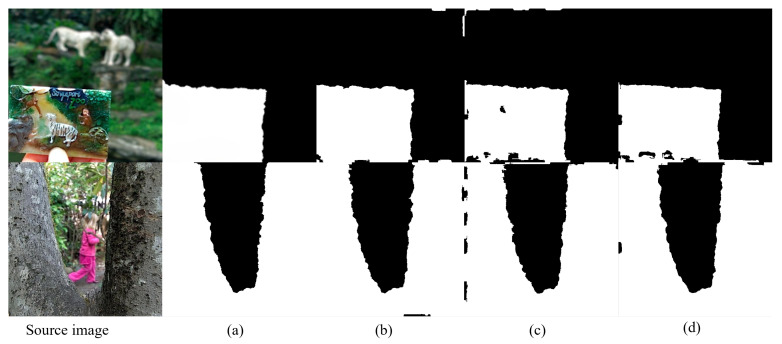
Comparison of decision maps generated by different loss functions. (**a**) with the complete loss function, (**b**) without edge alignment loss, (**c**) without mask loss, and (**d**) without quality loss generated using different loss function components.

To investigate the impact of different loss components on decision graph inference, we conduct a comparative analysis using representative samples from the Lytro dataset, as illustrated in Figure 9. The first column displays the foreground-focused source image, followed by four decision graph results. These comparisons demonstrate that KCUNet’s performance, which employs a high-fidelity fusion strategy guided by decision diagrams, is directly influenced by the accuracy of these decision diagrams. The experimental results demonstrate that removing $L_{EA}$ leads to blurred focus boundaries, as this loss function plays a crucial role in capturing subtle edge features through its combination of multiscale structural similarity and regional consistency constraints. Without it, the network struggles to accurately detect fine edge details. Similarly, the absence of $L_{Mask}$ results in increased misclassified pixels and coarse edges, since this component maintains structural smoothness and regional consistency in the decision graph. Its removal weakens global constraints on focus region delineation, producing irregular boundaries and scattered misclassified points. Eliminating $L_{Qg}$ causes a significant rise in decision graph noise, attributable to the loss of its gradient-aware mechanism that normally preserves high-frequency information. This degradation highlights its importance in processing texture-rich regions effectively. By contrast, when all loss components are incorporated, the decision graph achieves optimal performance, underscoring the complementary and essential nature of each term in ensuring high-quality results.

### 4.6. Hyperparameter Tuning Experiments

This section examines the influence of channel quantity and feature map size on the model’s fusion performance. To investigate these factors, three experimental configurations were designed:

(i) Progressive Channel Variation with Feature Map Scaling: In the encoder, the channel count progressively increases while the feature map dimensions are halved at each deeper layer. Conversely, in the decoder, the channel count gradually decreases, and the feature map size incrementally expands to restore the original dimensions.

(ii) Fixed Channel and Feature Map Configuration: Both the encoder and decoder maintain a constant channel count and feature map size throughout the network.

(iii) Constant Channel Count with Feature Map Scaling: The number of channels remains unchanged across all layers, while the encoder reduces the feature map size by half at each stage, and the decoder progressively upscales it to recover the original resolution.

**Figure 10:**
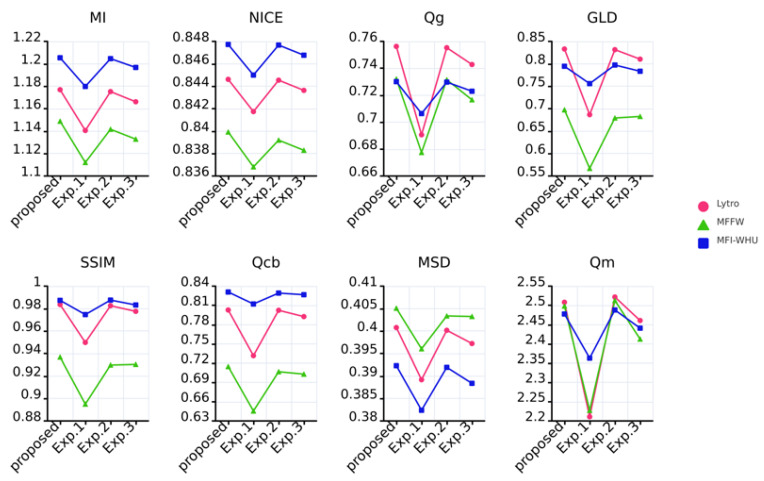
The values of the eight fusion metrics when fusing different datasets using KCUNet and the three sets of experimental models, respectively.

Table 4 presents the detailed parameters of input channel counts and feature map sizes for each encoder and decoder in the three experimental configurations. Figure 10 displays the values of eight fusion metrics obtained by applying KCUNet and the three experimental models to the Lytro, MFFW, and MFI-WHU datasets. In the figure, circles, triangles, and squares denote the fusion performance curves for the Lytro, MFFW, and MFI-WHU datasets, respectively. The results demonstrate that maintaining a constant feature map size yields superior performance compared to reducing it, suggesting that downscaling feature maps leads to information degradation. Additionally, dynamically adjusting the channel count while preserving the feature map size further enhances fusion performance. To assess the impact of maintaining a constant feature map size on computational complexity, we conduct a comparative analysis between the traditional downsampling architecture and the proposed method using the Lytro dataset. The experiments show that the average fusion time of the traditional downsampling method is 1.21 s, which is only 15.7% of that of our method (7.69 s), but its fusion quality metrics lag behind across the board: our method achieves a significant improvement in key metrics, such as the increase in MI from 1.170 to 1.177 (+0.6%), the increase in $Q_{G}$ from 0.747 to 0.756 (+1.2%), and the increase in $Q_{M}$ from 2.460 to 2.508 (+2.0%). These findings suggest that the enhanced capabilities in focus boundary detection and detail preservation come at the cost of reduced computational efficiency.

## 5. Conclusions

This paper introduces KCUNet, a novel deep learning model for MFIF. Built upon the U-Net architecture, KCUNet incorporates parallel connections between the KAN layer and the convolutional layer during the encoding stage to efficiently extract image features while maintaining constant spatial dimensions of feature maps. This design preserves high-resolution details while progressively increasing channel counts to capture multi-level feature representations. During the decoding phase, the model adopts an inverse strategy by progressively decreasing channel counts while maintaining constant feature map sizes. This approach enables focused processing and effective fusion of key features derived from the encoder. The integration of a CGA mechanism in the attention module enhances edge-related feature emphasis, effectively reducing DSE and preserving critical edge information. In addition, the proposed hybrid loss function comprehensively evaluates model performance across multiple aspects, including edge alignment, mask prediction, and fused image quality. Extensive comparative experiments with 15 SOTA methods demonstrate KCUNet’s superior performance in both qualitative and quantitative assessments. While achieving notable results in MFIF tasks, the model shows limitations in certain complex scenarios (e.g., strong defocus background effects and multi-source image fusion) and requires further improvement in computational efficiency. These aspects represent important directions for future research and model optimization.

In conclusion, KCUNet achieves significant progress in the MFIF domain by delivering high-quality, fully focused images. Its success stems from the integration of KAN with convolutional layers, an innovative attention module, and a carefully designed loss function. Future work will focus on enhancing the network’s efficiency, extending its application to other image processing tasks, and exploring advanced attention mechanisms for further performance gains.

## Figures and Tables

**Table 1:** Output channel specifications across model modules.

Encoder				S–CGANet				Decoder	
Eecoder 1	16	KAN layer	3	S–CGANet 1	16	Conv layer 1	16	Decoder 1-1	1
		3 × 3 Conv + 3 × 3 Conv	3						
						Conv layer 2	1	Decoder 1-2	16
		3 × 3 Conv + 1 × 1 Conv	16						
Eecoder 2	32	KAN Layer	16	S–CGANet 2	32	Conv layer 1	32	Decoder 2-1	1
		3 × 3 Conv + 3 × 3 Conv	16						
						Conv layer 2	1	Decoder 2-2	32
		3 × 3 Conv + 1 × 1 Conv	32						
Eecoder 3	48	KAN Layer	32	S–CGANet 3	48	Conv layer 1	48	Decoder 3-1	1
		3 × 3 Conv + 3 × 3 Conv	32						
						Conv layer 2	1	Decoder 3-2	48
		3 × 3 Conv + 1 × 1 Conv	48						
Eecoder 4	64	KAN Layer	48	/				Decoder 4-1	1
		3 × 3 Conv + 3 × 3 Conv	48						
								Decoder 4-2	64
		3 × 3 Conv + 1 × 1 Conv	64						

**Table 2:** Average scores of the fused images of each algorithm for Lytro, MFFW, and MFI-WHU datasets. The red number is the best, the blue number is the second best, and the green number is the third best.

(a) Lytro									
	MI	NCIE	$Q_{G}$	GLD	SSIM	$Q_{\mathrm{CB}}$	MSD	$Q_{M}$	Time (s)
BFMF [22]	1.120786	0.841565	0.748662	0.829980	0.977915	0.797808	0.397188	2.056438	0.82
DCT-EOL [48]	1.135365	0.841971	0.750989	0.825581	0.975609	0.795957	0.397804	2.208693	0.34
CNN [8]	1.109687	0.840168	0.751597	0.832701	0.978043	0.799881	0.396286	2.041220	89.34
DRPL [10]	1.091545	0.838783	0.752593	0.839841	0.981742	0.794202	0.396623	1.702978	0.29
ECNN [49]	1.127325	0.841359	0.747771	0.818095	0.975674	0.795808	0.396967	2.200383	72.85
FusionDiff [54]	1.133919	0.841958	0.751038	0.825959	0.975511	0.795062	0.397590	2.212881	5.63
IFCNN [25]	0.926848	0.829266	0.725188	0.802673	0.951572	0.723420	0.382435	1.185922	0.08
MFF-GAN [37]	0.804653	0.823709	0.659247	0.712464	0.882123	0.645681	0.378113	0.641194	1.06
MFIF-GAN [32]	1.131316	0.841718	0.752563	0.830668	0.979351	0.800559	0.398151	2.179960	1.26
MiT [52]	1.149638	0.842643	0.750932	0.831739	0.968942	0.785913	0.398152	2.36881	2.89
Swin Fusion [51]	1.153513	0.841352	0.753391	0.840163	0.981411	0.793856	0.399057	2.17592	1.62
SESF [11]	1.102258	0.839721	0.746465	0.825042	0.975765	0.796275	0.394775	2.094775	0.31
U2Fusion [50]	0.772468	0.822085	0.609294	0.665709	0.791230	0.568214	0.399414	0.498487	2.57
MMF-Net [30]	0.971946	0.832144	0.722871	0.81261	0.951421	0.750805	0.383743	1.39292	0.51
ZMFF [50]	0.883846	0.827076	0.703035	0.785289	0.931368	0.741161	0.391849	0.599906	359.26
Proposed	1.176907	0.844614	0.756260	0.832972	0.983603	0.802801	0.400774	2.508082	7.69
**(b) MFFW**									
	**MI**	**NCIE**	$Q_{G}$	**GLD**	**SSIM**	$Q_{\mathrm{CB}}$	**MSD**	$Q_{M}$	**Time (s)**
BFMF [22]	0.781535	0.820600	0.618944	0.557555	0.932778	0.680319	0.366949	0.471419	1.37
DCT-EOL [48]	0.736733	0.818880	0.623827	0.539449	0.763540	0.665272	0.357793	0.496458	0.56
CNN [8]	0.767708	0.819903	0.627882	0.563330	0.782654	0.675026	0.364013	0.476062	119.71
DRPL [10]	0.826484	0.822331	0.680520	0.646220	0.840019	0.695705	0.374099	0.796502	0.74428
ECNN [49]	0.744842	0.819179	0.622812	0.547708	0.759878	0.669305	0.359607	0.495281	102.47
FusionDiff [54]	1.074970	0.819279	0.722778	0.672428	0.872219	0.704530	0.359923	1.944150	6.87
IFCNN [25]	0.750173	0.819172	0.606258	0.526317	0.758045	0.626119	0.361500	0.393533	0.13
MFF-GAN [37]	0.706532	0.817821	0.571507	0.480930	0.712813	0.584129	0.367098	0.341773	1.71
MFIF-GAN [32]	0.771574	0.820045	0.630538	0.571361	0.790328	0.688156	0.364516	0.492021	1.88
MiT [52]	0.945537	0.821366	0.718129	0.728854	0.901684	0.702352	0.364124	2.28531	4.37
SwinFusion [51]	1.016355	0.831712	0.712634	0.695388	0.924638	0.703384	0.365321	2.15374	1.83
SESF [11]	0.738324	0.818898	0.624714	0.551459	0.827208	0.673302	0.354221	0.474540	0.74
U2Fusion [50]	0.697070	0.817080	0.536063	0.469957	0.667186	0.549475	0.389798	0.311336	3.94
MMF-Net [30]	0.815816	0.821902	0.683651	0.645986	0.838008	0.690607	0.372084	0.763692	1.15
ZMFF [50]	0.763981	0.819897	0.654051	0.623221	0.845644	0.6751245	0.373213	0.478555	373.33
Proposed	1.148829	0.839920	0.732071	0.697461	0.936793	0.714825	0.405085	2.498152	8.87
**(c) MFI-WHU**									
	**MI**	**NCIE**	$Q_{G}$	**GLD**	**SSIM**	$Q_{\mathrm{CB}}$	**MSD**	$Q_{M}$	**Time (s)**
BFMF [22]	1.162822	0.844579	0.726402	0.79841	0.986869	0.831481	0.387028	2.267257	1.03
DCT-EOL [48]	1.17627	0.844935	0.722151	0.761314	0.979879	0.811306	0.390087	2.384231	0.39
CNN [8]	1.15616	0.844016	0.729326	0.799211	0.98745	0.831087	0.386453	2.256109	92.52
DRPL [10]	1.089268	0.838904	0.725469	0.793622	0.985109	0.823178	0.382771	1.58352	0.56
ECNN [49]	1.177014	0.845299	0.729397	0.794453	0.987218	0.830274	0.388292	2.380926	83.34
FusionDiff [54]	1.161053	0.844275	0.729286	0.798314	0.987122	0.831801	0.386809	2.304451	7.25
IFCNN [25]	0.897787	0.8278	0.694425	0.781341	0.957726	0.740476	0.363874	1.002306	0.22
MFF-GAN [37]	0.757389	0.82176	0.638063	0.716602	0.885304	0.636203	0.351768	0.526223	1.41
MFIF-GAN [32]	1.167011	0.845152	0.724862	0.782565	0.982538	0.82458	0.388139	2.318145	1.09
MiT [52]	1.162259	0.837136	0.726149	0.796272	0.981222	0.811981	0.388731	2.38185	3.53
SwinFusion [51]	1.089268	0.838904	0.725469	0.793622	0.985102	0.823178	0.389668	1.58352	1.73
SESF [11]	1.14944	0.843643	0.72259	0.795414	0.985044	0.823807	0.387253	2.269356	0.67
U2Fusion [50]	0.679615	0.818677	0.543644	0.615793	0.758541	0.513538	0.366102	0.340131	3.17
MMF-Net [30]	1.06377	0.837174	0.723202	0.796456	0.983624	0.818679	0.38084	1.373467	0.59
ZMFF [50]	0.775562	0.822346	0.633181	0.693681	0.677882	0.713661	0.361973	0.370791	389.21
Proposed	1.205382	0.847725	0.730077	0.794514	0.987327	0.830858	0.392306	2.477546	7.97

**Table 3:** Average scores of the fused images of each algorithm for Lytro, MFFW, and MFI-WHU datasets. The red number is the best, the yellow number is the second best, and the green number is the third best.

		MI	NCIE	$Q_{G}$	GLD	SSIM	$Q_{\mathrm{CB}}$	MSD	$Q_{M}$
Lytro	Proposed	1.176907	0.844614	0.756260	0.832972	0.983603	0.802801	0.400774	2.508082
	No KAN	1.171163	0.844148	0.744605	0.816912	0.978640	0.793031	0.398791	2.411444
	Difference	0.005744	0.000466	0.011655	0.016060	0.004962	0.009771	0.001983	0.096637
MFFW	Proposed	1.148829	0.839920	0.732071	0.697461	0.936793	0.714825	0.405085	2.498152
	No KAN	1.146606	0.839539	0.725526	0.684955	0.934455	0.705403	0.403914	2.453557
	Difference	0.002223	0.000381	0.006545	0.012506	0.002339	0.009422	0.001171	0.044595
MFI-WHU	Proposed	1.205382	0.847725	0.730077	0.794514	0.987327	0.830858	0.392306	2.477546
	No KAN	1.200248	0.846946	0.725282	0.789917	0.984802	0.827545	0.389669	2.437547
	Difference	0.005134	0.000779	0.004795	0.004597	0.002525	0.003313	0.002637	0.040000

**Table 4:** Parameters specific to each encoder and decoder, including input channel count and size of input feature maps. H and W are the feature map height and width, respectively.

Experiment	Parameter	Encoder1	Encoder2	Encoder3	Encoder4	Decoder4	Decoder3	Decoder2	Decoder1
Proposed	Channels	3	16	32	48	64	48	32	16
	Size	H,W	H,W	H,W	H,W	H,W	H,W	H,W	H,W
Experiment1	Channels	3	16	32	48	64	48	32	16
	Size	H,W	H/2,W/2,	H/4,W/4	H/8,W/8	H/8,W/8	H/4,W/4	H/2,W/2	H,W
Experiment2	Channels	3	3	3	3	3	3	3	3
	Size	H,W	H,W	H,W	H,W	H,W	H,W	H,W	H,W
Experiment3	Channels	3	3	3	3	3	3	3	3
	Size	H,W	H/2,W/2,	H/4,W/4	H/8,W/8	H/8,W/8	H/4,W/4	H/2,W/2	H,W

## Data Availability

The original data used during this study are included in the article. Further inquiries can be directed to the corresponding author.
